# A Widespread Distribution of Genomic CeMyoD Binding Sites Revealed and Cross Validated by ChIP-Chip and ChIP-Seq Techniques

**DOI:** 10.1371/journal.pone.0015898

**Published:** 2010-12-29

**Authors:** Haiyan Lei, Tetsunari Fukushige, Wei Niu, Mihail Sarov, Valerie Reinke, Michael Krause

**Affiliations:** 1 National Institute of Diabetes and Digestive and Kidney Diseases, National Institutes of Health, Bethesda, Maryland, United States of America; 2 Department of Molecular, Cellular Developmental Biology, Yale University, New Haven, Connecticut, United States of America; 3 Max Planck Institute for Molecular Cell Biology and Genetics, Dresden, Germany; 4 Department of Genetics, Yale University School of Medicine, New Haven, Connecticut, United States of America; Istituto Dermopatico dell'Immacolata, Italy

## Abstract

Identifying transcription factor binding sites genome-wide using chromatin immunoprecipitation (ChIP)-based technology is becoming an increasingly important tool in addressing developmental questions. However, technical problems associated with factor abundance and suitable ChIP reagents are common obstacles to these studies in many biological systems. We have used two completely different, widely applicable methods to determine by ChIP the genome-wide binding sites of the master myogenic regulatory transcription factor HLH-1 (CeMyoD) in *C. elegans* embryos. The two approaches, ChIP-seq and ChIP-chip, yield strongly overlapping results revealing that HLH-1 preferentially binds to promoter regions of genes enriched for E-box sequences (CANNTG), known binding sites for this well-studied class of transcription factors. HLH-1 binding sites were enriched upstream of genes known to be expressed in muscle, consistent with its role as a direct transcriptional regulator. HLH-1 binding was also detected at numerous sites unassociated with muscle gene expression, as has been previously described for its mouse homolog MyoD. These binding sites may reflect several additional functions for HLH-1, including its interactions with one or more co-factors to activate (or repress) gene expression or a role in chromatin organization distinct from direct transcriptional regulation of target genes. Our results also provide a comparison of ChIP methodologies that can overcome limitations commonly encountered in these types of studies while highlighting the complications of assigning in vivo functions to identified target sites.

## Introduction

Chromatin immunoprecipitation (ChIP) is becoming an increasingly popular means to survey the genome-wide distribution of chromatin-associated proteins and their post-translational modifications. This approach is challenging when applied to many transcription factors because they are often present at low levels within cells and are located discretely, and often infrequently, within chromatin. These problems are amplified in complex tissues if the transcription factor is expressed in only a subset of the cell types within the tissue/organism or is temporally restricted. In addition, many transcription factors lack specific antibodies suitable for ChIP, leaving researchers to opt for indirect methods to interrogate chromatin-associated binding site for the factor of interest.

To address these issues, we have compared two completely different ChIP methods to determine the genome-wide distribution of HLH-1 (sometimes referred to as CeMyoD) binding sites in *C. elegans* mixed stage embryos. One method utilized vast over-production of HLH-1 from a heat shock inducible transgene, immunoprecipitation with an anti-HLH-1 antibody, and hybridization to a genome tiling array (ChIP-chip). The second method employed a Green Fluorescent Protein (GFP)-tagged, low copy number, integrated *hlh-1* translational fusion transgene, immunoprecipitation with an anti-GFP antibody, and next-generation sequencing (ChIP-seq). The advantage of over expression is that it can overcome limitations in transcription factor abundance and antibody affinity. The obvious disadvantage of over expression is the potential for ectopic binding to result in misleading profiles of the genome wide distribution. This latter concern may also be applicable to certain epitope-tagged transgenes, although likely less problematic when expression is directed by sequences representing the natural genomic context for the gene of interest that are integrated as one or a few copies. In fact, early results from GFP-tagged coding regions using bacterial recombineering in fosmid clones look very promising in terms of recapitulating endogenous patterns of transcription factor expression and binding [1], [2]. We reasoned that the use of two very different approaches to determine the genome-wide HLH-1 binding sites would allow us to cross validate the data from each while exploring techniques that could be used to overcome limitations that will be commonly encountered in the study of transcription factor binding sites by ChIP.

The results from each experimental approach were remarkably similar and suggested that there are a large number of HLH-1 binding sites genome-wide, primarily located in promoter regions of candidate target genes. Analysis of these HLH-1-positive ChIP genomic fragments demonstrated that E-box (CANNTG) sequences, known binding sites for HLH-1 and related helix-loop-helix factors, are the predominantly over-represented motif. We have validated our HLH-1 candidate binding sites by ChIP-qPCR, comparisons to independently derived ChIP data, and by reporter gene expression. This analysis demonstrates that both over expression and epitope-tagging of transcription factors can provide a valid profile of DNA binding sites throughout the genome. Moreover, our results highlight the difficulty in ascribing biological functions to ChIP-defined binding sites, a problem common to all current approaches.

## Results

Striated muscle cell fate specification and differentiation in animals is orchestrated at the transcriptional level by Myogenic Regulatory Factors (MRFs) [3], [4]. HLH-1 is the lone MRF homolog in *C. elegans* and it functions together with other transcription factors to specify bodywall muscle fate and direct differentiation [5], [6]. It is generally assumed that HLH-1 directly activates muscle-specific gene expression, just as MRFs act as transcriptional activators of muscle genes in higher eucaryotes. In vitro, HLH-1 binds the canonical basic helix-loop-helix DNA site known as an E-box (CANNTG). However, this simple sequence is present frequently throughout the genome (291,374 sites in *C. elegans*), requiring additional experimental evidence to directly link HLH-1 to downstream target gene activation. Chromatin immunoprecipitation (ChIP), coupled with either whole genome tiling arrays (ChIP-chip) or high throughput sequencing (ChIP-seq), offers the potential to make this link and to provide insight into the molecular mechanisms and logic underlying myogenesis and other differentiation programs.

Polyclonal antibodies have been prepared against full-length HLH-1 fusion proteins in chickens. Previous attempts to use this antibody in chromatin immunoprecipitation experiments in wild type animals to assay binding to cis-acting *hlh-1* enhancer fragments known to be involved in positive auto-regulation were unsuccessful [7]. We also failed to detect HLH-1 binding to autoregulatory enhancer sequences in strains harboring high copy number *hlh-1* reporter transgenes. The inability to ChIP endogenous levels of HLH-1 reflects a problem likely to be common for transcription factor studies in many model systems, leading us to explore alternative approaches to profile HLH-1 binding sites on a genomic scale. HLH-1 is an excellent test case for this approach because muscle gene expression has been well documented in *C. elegans*, providing a predictable set of target genes that should be identified by ChIP if HLH-1 function is direct and restricted to bodywall muscle genes.

### HLH-1 binds to the promoter region of 1,000s of potential target genes

We have previously described an integrated transgene expressing a full-length *hlh-1* cDNA from a heat shock promoter [7]. Heat shock induction of HLH-1 during early embryogenesis results in high levels of nuclear-localized protein and widespread conversion of blastomeres to the bodywall muscle fate, demonstrating this over expressed factor is functional and can activate target genes in muscle. We have used this strain to over express HLH-1 in mixed stage embryos that were collected three hours post-induction for ChIP using an anti-HLH-1 antibody raised in chickens (see Materials & Methods). ChIP-enriched DNA sequences were amplified and hybridized to high-density DNA tiling arrays spanning the entire *C. elegans* genome (Nimblegen) with a probe length of 50 bp, centered 36 bp apart. Regions of HLH-1 genomic binding were determined using CARPET [8] and annotated to the nearest transcriptional start site (TSS).

**Figure 1:**
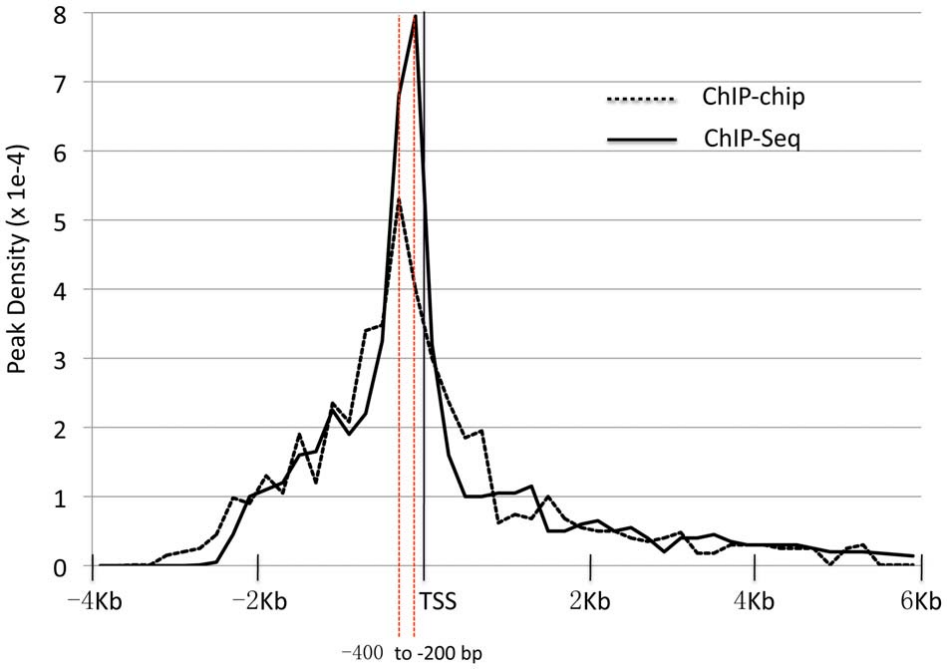
The distribution of HLH-1 ChIP peaks relative to the transcriptional start site (TSS) of protein coding genes. The density of peaks (10^−4^) located within 2 kb upstream or 6 kb downstream of defined TSS (WS195) were binned in sets of 200 bp, averaged and the value plotted. Similar distributions of HLH-1 binding sites were observed for both ChIP-chip (dashed) and ChIP-seq (solid) datasets. Maximal clustering of binding sites was observed in the −200 to −400 bp bins with a sharp decline upstream of the gene and gradual decline throughout the region downstream of the TSS.

HLH-1 ChIP-chip data from mixed stage embryo populations was collected from three independent, biological replicates. The data was normalized using the Bi-weight and Mean Summarization functions in CARPET [8] revealing correlation coefficients between 0.71 and 0.78 for all replicate comparisons. We found 1,376 HLH-1 bound peaks genome wide (Table S1) with bound interval sequences averaging 1,454 bp in length. The majority of HLH-1 peaks (82%) were located in the promoter or intragenic regions relative to protein coding genes; the remaining peaks (18%) were located in more distal regions (>2 Kb from the TSS). The distribution of HLH-1 bound intervals relative to the TSS revealed the −200 to −400 bp region as the most frequent location of binding sites (Figure 1) and identified 1,032, of the predicted ∼20,000 (WS195) protein encoding genes, as potential targets of HLH-1 regulation (Table S2).

As part of the modENCODE pipeline, a GFP-tagged *hlh-1* translational fusion transgene was generated by recombineering as previously described [1], [2]. This transgene was integrated into the genome in low copy number by particle bombardment using a standardized modENCODE protocol for targeted transcription factors that aims to reflect the natural temporal and spatial pattern of expression for the factor of interest with expression at, or near, wild type levels. Mixed staged, transgenic embryos were isolated for chromatin preparation from two biological replicates and subjected to ChIP-seq as previously described [1].

**Figure 2:**
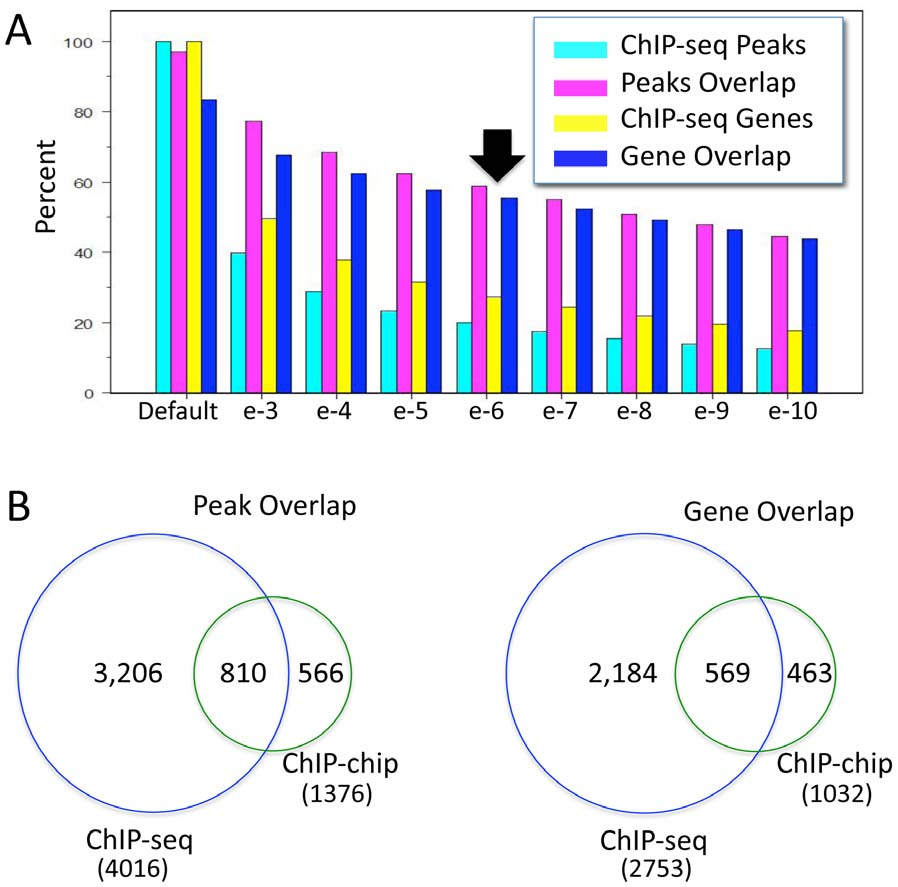
The overlap of HLH-1 ChIP peak and gene calls. A) Peaks, or genes associated with peaks, from ChIP-seq data using the default modENCODE thresholds were used to normalize the overlapping number of peaks or genes in the ChIP-chip data. The percent overlap in peak or gene lists between the ChIP-chip data compared to the ChIP-seq data at default settings or at increasing peak call stringency using p-values for the ChIP-seq data is plotted. The peak and gene numbers associated with the 10^−6^ threshold (arrow) was chosen as a more stringent cutoff for subsequent analysis. B) Proportional area Venn diagrams showing the overlap of peak (left) or gene (right) calls from the ChIP-seq (10^−6^ threshold) compared to the ChIP-chip data.

Using the CARPET package [8] to analyze modENCODE ChIP-seq data, we found 20,143 peaks of HLH-1 binding associated with 10,037 candidate genes. Because this analysis linked HLH-1 to nearly half of all known protein coding genes in *C. elegans*, we applied increasingly stringent thresholds for peak calls (using p-values) to further restrict the genes used for our analysis (Figure 2A). At a p-value cutoff between 10^−5^ and 10^−6^, we noted an inflection point in the data where the peak and gene values for the two ChIP datasets transitioned from a non-linear to a near linear relationship. Similarly, the overlaps between ChIP-seq gene calls and previously reported bodywall muscle expressed genes (described below) were near linear at cutoff values between 10^−5^ and 10^−10^. This suggested that at stringencies between 10^−6^ and 10^−10^, the reduction in peaks and genes identified was reflecting a simple truncation of the number of calls rather than increasing, biologically relevant discrimination. Thus, we chose a conservative cutoff peak p-value at 10^−6^ for all subsequent analysis of the ChIP-seq data, resulting in 4,016 peaks associated with 2,753 unique candidate target genes (Tables S2 & S3) with an average peak interval length of 534 bp. The distribution of peak location relative to the TSS for this set of genes is shown in Figure 1. As with the HLH-1 ChIP-chip data, the most frequent location of the HLH-1 ChIP-seq peaks (10^−6^ cutoff) was just upstream of the TSS. Data from either method had a distribution that fell off steeply upstream of the TSS, but remained elevated above background downstream of the TSS, extending nearly 6 Kb.

We employed the CARPET package [8] Com&Easy function and Galaxy [9] to further compare the overlap of peak calls between HLH-1 ChIP datasets (see Materials and Methods). Of the 1,376 peaks identified after heat shock induction of HLH-1, 59% overlapped with the ChIP-seq peak calls (10^−6^ cutoff) (Figure 2B.). Software developed by Lund and colleagues (http://elegans.uky.edu/MA/progs/Compare.html) was used to compare the lists of genes associated with peaks in each experimental approach. Comparison of gene lists revealed that 569 of the 1,032 (55%) candidate genes associated with ChIP-chip were present in the ChIP-seq (10^−6^ cutoff) list of 2,753 genes (Figure 2B, Table S2); the overlap increased to 862 (84%) genes for the default ChIP-seq data. Visual inspection of the heat shock versus transgene HLH-1 ChIP datasets in the Integrated Genome Browser (IGB) confirmed substantial overlap of the patterns (Figure S1). We noted that the 10^−6^ ChIP-seq data failed to identify many ChIP-chip genes (45%), a result that likely reflects not only the more stringent cutoff threshold, but also several technical differences between the methods. For example, the two ChIP methods used different antibodies for immunoprecipitation (chicken anti-HLH versus donkey anti-GFP) that likely cross-react differently with chromatin proteins resulting in very different non-specific background binding sites. However, the HLH-1 binding sites common to these two very different methods suggest that many (55–84%) of the gene calls reflected bona fide regions of HLH-1 localization in embryonic chromatin.

### HLH-1 ChIP peaks validate in vitro

**Figure 3:**
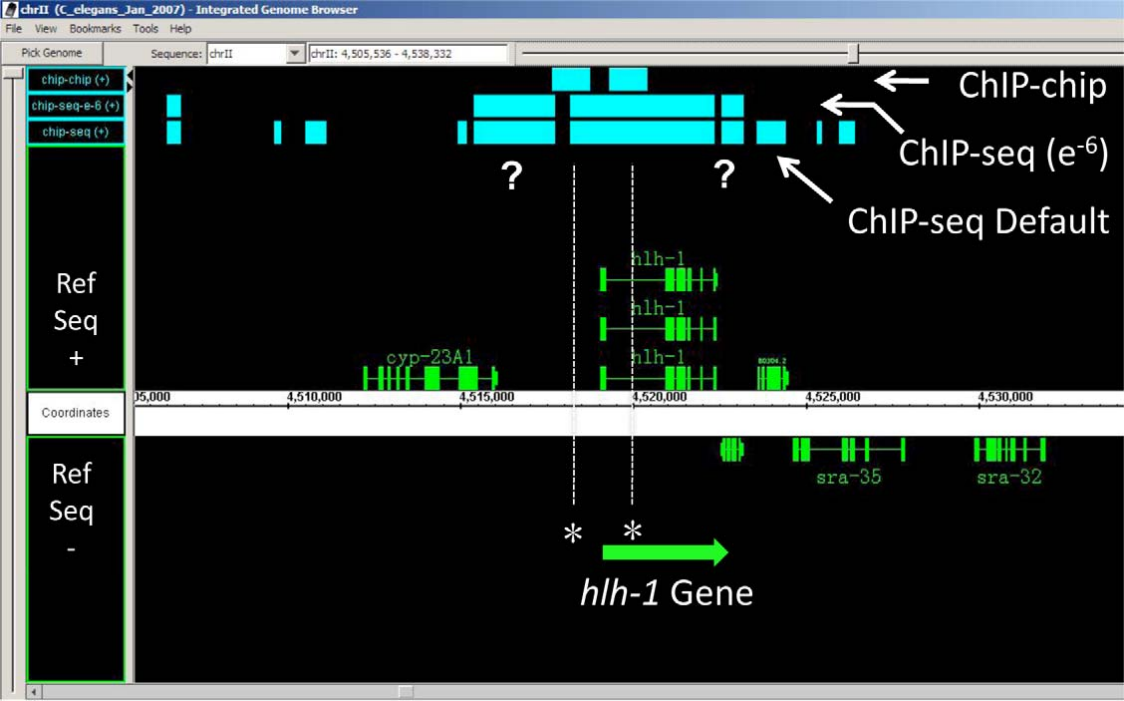
Visualization of HLH-1 ChIP data at the *hlh-1* locus. The *hlh-1* gene positively autoregulates its own expression through several previously defined enhancers located upstream and within the first intron of the gene [7]. An Integrated Genome Browser (IGB) view of the ChIP data centered on the *hlh-1* locus on LGII of *C. elegans* shows the peak intervals at top (blue boxes) relative to gene coding regions on the plus and minus coding strands below (green boxes). ChIP-seq intervals using both default and 10^−6^ thresholds are shown for comparison to the ChIP-chip intervals; both approaches identify previously defined HLH-1 binding sites (*) involved in autoregulation. The ChIP-seq data suggest additional binding sites might exist (?) further upstream and downstream of the *hlh-1* gene.

Previous ChIP studies have characterized HLH-1 bound enhancer regions regulating *hlh-1*, providing a very limited, yet important, internal control set of sites for comparisons to our most recent data. HLH-1 positive auto-regulation of its own expression occurs through several enhancer elements located upstream of the coding region and within the first large intron of *hlh-1* [7]. Comparison of both the ChIP-chip and ChIP-seq (10^−6^) HLH-1 data to these known sites demonstrates that all four previously described HLH-1 bound enhancers were detected by both genome-wide approaches (Figure 3), with the ChIP-seq data suggesting that additional HLH-1 binding sites may extend further upstream of the gene than previously appreciated. A second previously characterized HLH-1 binding site upstream of the gene *unc-120* failed to be detected by ChIP-chip, but was detected by ChIP-seq using 10^−6^ thresholding (data not shown).

**Figure 4:**
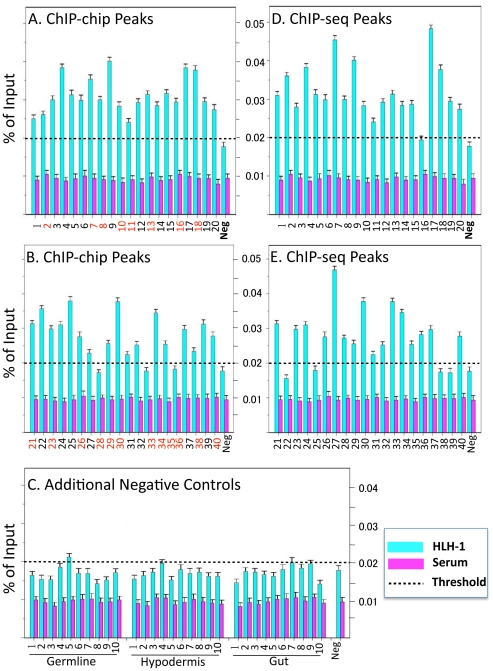
Quantitative PCR validation of ChIP data. (A, B) ChIP-chip peak validation. Amplicons corresponding to HLH-1 ChIP-chip peaks ranked at the top (A; 1–20), middle (B; 21–30) or bottom (B; 31–40) of the list were selected for validation. These amplicons were tested in a minimum of two independent ChIP-qPCR experiments each for enrichment with HLH-1 antibody (blue) over pre-immune serum (purple). Using a validation threshold of greater than 2-fold enrichment over pre-immune serum alone (dotted line), 37/40 amplicons validated by qPCR whereas a negative control region (Neg) did not. Of the 40 ChIP-chip amplicons tested, 20 also corresponded to ChIP-seq peak calls (A & B black numbers); all 20 of these validated by ChIP-qPCR. (C) For each of 10 genes previously described as expressed in the germline, hypodermis, or intestinal cells, amplicons were chosen from the putative promoter regions upstream of the TSS. These 30 amplicons, along with the *hlh-1* gene negative control regions, were assayed for enrichment after HLH-1 ChIP (blue) relative to pre-immune serum (purple). Only one of the 30 negative controls crossed the validation threshold of 2-fold enrichment over background. (D, E) ChIP-seq peak validation. Amplicons corresponding to HLH-1 ChIP-seq peaks not detected by ChIP-chip and ranked at the top (D; 1–20), middle (E; 21–30) or bottom (E; 31–40) of the list were selected for validation. From the top ranked peak amplicons, 19/20 validated, as did 8/10 for each of lower ranked peaks. Amplicon information, primer sequences, and additional negative controls are given in Table S4.

Quantitative PCR (qPCR) was used to confirm HLH-1 bound regions determined by either ChIP-chip or ChIP-seq methods. For the HLH-1 ChIP-chip peak calls, we generated qPCR amplicons to interrogate 20 regions ranked in the top 300 by peak score, 10 peaks ranked in the middle between 301–800, and 10 peaks ranking at the bottom between 801–1,376. As shown in Figure 4, results from two separate HLH-1 ChIP-qPCR experiments confirmed the ChIP-chip results for 20/20 top-ranked peaks (Figure 4A), 9/10 middle-ranked peaks, and 8/10 low-ranked peaks (Figure 4B). Taken together, the ChIP qPCR results confirmed 93% of sampled peaks identified by ChIP-chip. Of the 40 ChIP-chip amplicons assayed, 20 (50%) corresponded to ChIP-seq peaks (Figure 4A & B, black numbers); all 20 were positive by qPCR, confirming these HLH-1 binding sites by three independent methods. As negative controls for ChIP-qPCR, we used a region from the *hlh-1* promoter previously shown to be unable to preferentially bind HLH-1 [7] (Figure 4) and amplicons upstream of genes previously identified as being expressed and/or enriched in germ line [10], hypodermis (http://tock.bcgsc.bc.ca/cgi-bin/sage140), or gut [11], [12], [13]. HLH-1 ChIP-qPCR demonstrated binding slightly above background for only one of these 31 negative control amplicons (Figure 4C). These results suggested that the vast majority of HLH-1 bound intervals detected in embryonic chromatin by array hybridizations are factor-specific and are reproducibly detected by deep sequencing or qPCR, with the confidence of detection directly related to the peak score.

As noted above, the ChIP-seq (10^−6^) data revealed a number of peaks that were not present in the ChIP-chip data. To determine if these potential sites of HLH-1 binding were valid, we assayed 20 amplicons from ChIP-seq peaks with scores from the top 1,000 as well as 10 amplicons each from peaks scoring in the middle or bottom ranks. As shown in Figure 4D & E, all but one of the 20 top peaks validated as did 80% of the peaks from the middle or bottom ranked lists. Importantly, the method used to validate the ChIP-seq data was immunoprecipitation with anti-HLH-1 antibodies from mixed stage embryos over expressing the HLH-1, that is, our ChIP-chip protocol. We concluded that the vast majority of ChIP-seq peaks (10^−6^) were valid sites of HLH-1 binding and that our ChIP-chip data under-represented these sites.

### HLH-1 ChIP bound intervals contain E-Box sequence motifs and are enriched for muscle expression

**Figure 5:**
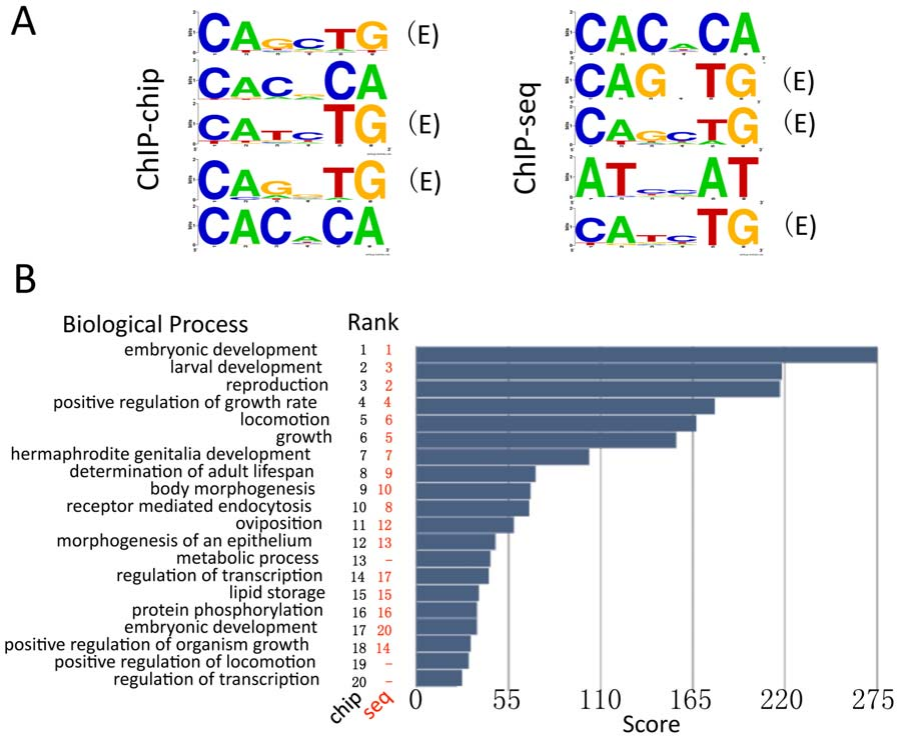
Over represented motifs and GO terms in HLH-1 ChIP data. A) Genomic sequences corresponding to the top 40 peaks of HLH-1 binding by either ChIP-chip or ChIP-seq (10^−6^ threshold) were analyzed for over represented motifs using MDscan and LOGO software (http://weblogo.berkeley.edu/logo.cgi). Shown are five representatives LOGOs of the top 10 sequence motifs returned for each dataset using a window width of six base pairs. An E-box (CANNTG; marked (E)), the preferred HLH-1 binding site, is a frequent hit (7/10) among the top motifs identified, regardless of ChIP method. The simple repeat CACACA (and its compliment) is also returned and has been previously associated with PHA-4 bound promoter sequences [1]. B) Gene Ontology (GO) analysis of genes identified by HLH-1 ChIP. The top 20 Biological Process (BP) GO term hits for the 1,032 genes identified by HLH-1 ChIP-chip are shown in rank order. Seventeen of the 20 GO BP terms were also returned by a similar analysis of the 2,753 genes identified by HLH-1 ChIP-seq (10^−6^ threshold) with nearly identical rank order. Most terms are consistent with embryonic growth and muscle development, including the terms oviposition and hermaphrodite genitalia development that include many genes common to general processes active in embryogenesis.

To determine if HLH-1 bound peaks identified by our two different ChIP methods contained over represented motifs, DNA sequences corresponding to HLH-1-bound genomic intervals were analyzed using MDscan [14]. Analysis of the sequences from the top 40 peak scores from HLH-1 ChIP-chip revealed E-Box sequences (CANNTG) as the primary over represented sequence motif when the width interval was set to either six or eight nucleotides; 7 of the top 10 motifs were E-boxes (Figure 5A). The same was true for the top 40 HLH-1 ChIP-seq peaks. In fact, E-Box sequences continued to be a top motif returned by MDscan analysis for peak scores extending to include the top 370 out of 1,376 HLH-1 ChIP-chip or top 120 out of 4,016 HLH-1 ChIP-seq intervals analyzed. The only other recurrent motif to emerge from the top ChIP binding sites was CACNCA (an its compliment), a motif common to many promoter regions (M. Krause, unpublished), and present in both the ChIP-chip and ChIP-seq datasets.

While E-boxes are a known consensus basic helix-loop-helix transcription factor binding motif and HLH-1 binds these sequences promiscuously in vitro [15], [16], [17], only a small subset of all possible E-boxes genome wide were identified by ChIP. Of the 291,374 E-box sequences present in the *C. elegans* genome, only 10,082 (3.5%) were identified after over expression of HLH-1 in our ChIP-chip experiments. Similarly, only 10,543 (3.6%) E-box sites were identified by ChIP-seq (10^−6^), a number that rises to 24,061 (8.3%) in the unabated, default ChIP-seq data. We concluded from this data that there is a high degree of selectivity for HLH-1 binding that restricts it to only a small fraction of the potential E-box sites present in the genome.

Gene Ontology (GO) analysis in GENECODIS [18], [19] and DAVID Bioinformatics Resources 6.7 [20], [21] was used to determine biological processes enriched in the candidate HLH-1 target gene lists. Enriched GO terms for the HLH-1 ChIP-chip peaks included embryonic development, larval development, reproduction, regulation of growth rate, locomotion, growth, and body morphogenesis (Figure 5B), consistent with the role of HLH-1 in embryonic development and bodywall myogenesis. Inspection of genes identified with unexpected GO terms, such as oviposition or hermaphrodite genitalia development, revealed they encoded factors for general functions common to many developmental processes, including cell division, cell migration, vesicular trafficking, and chromatin reorganization. The GO analysis of the HLH-1 ChIP-seq candidate target gene list was nearly identical, including the rank order of the relative processes that were enriched. A random set of 3,000 genes failed return the same processes when subjected to an identical GO analysis, suggesting HLH-1 candidate target genes identified by either approach were factor-, and not method-, specific.

If HLH-1 is involved in the direct activation of genes expressed in bodywall muscle, there should be a correlation between the HLH-1 target genes identified by ChIP and the bodywall muscle transcriptome. Several groups, using a variety of techniques and approaches, have profiled bodywall muscle gene expression in *C. elegans*. Examples include 1,064 genes enriched in bodywall muscles of L1 larvae using polyA binding protein tagging [22], and genes expressed in embryonic bodywall muscle based on reporter gene sorted blastomeres analyzed by microarray techniques [23] or SAGE [24]. We have also previously profiled the temporal pattern of genome wide expression in response to the heat shock induced *hlh-1* transgene used for ChIP-chip in this study [5], [6]; taking genes identified as up-regulated by *hlh-1* over expression 6 hours after induction resulted in a list of 927 genes. Finally, a compilation of community data at WormAtlas (http://www.wormatlas.org) was used to generate a list of 480 genes expressed in bodywall muscle that was further culled of genes also expressed in the hypodermis to yield a more tissue restricted list of 297 bodywall muscle genes.

**Figure 6:**
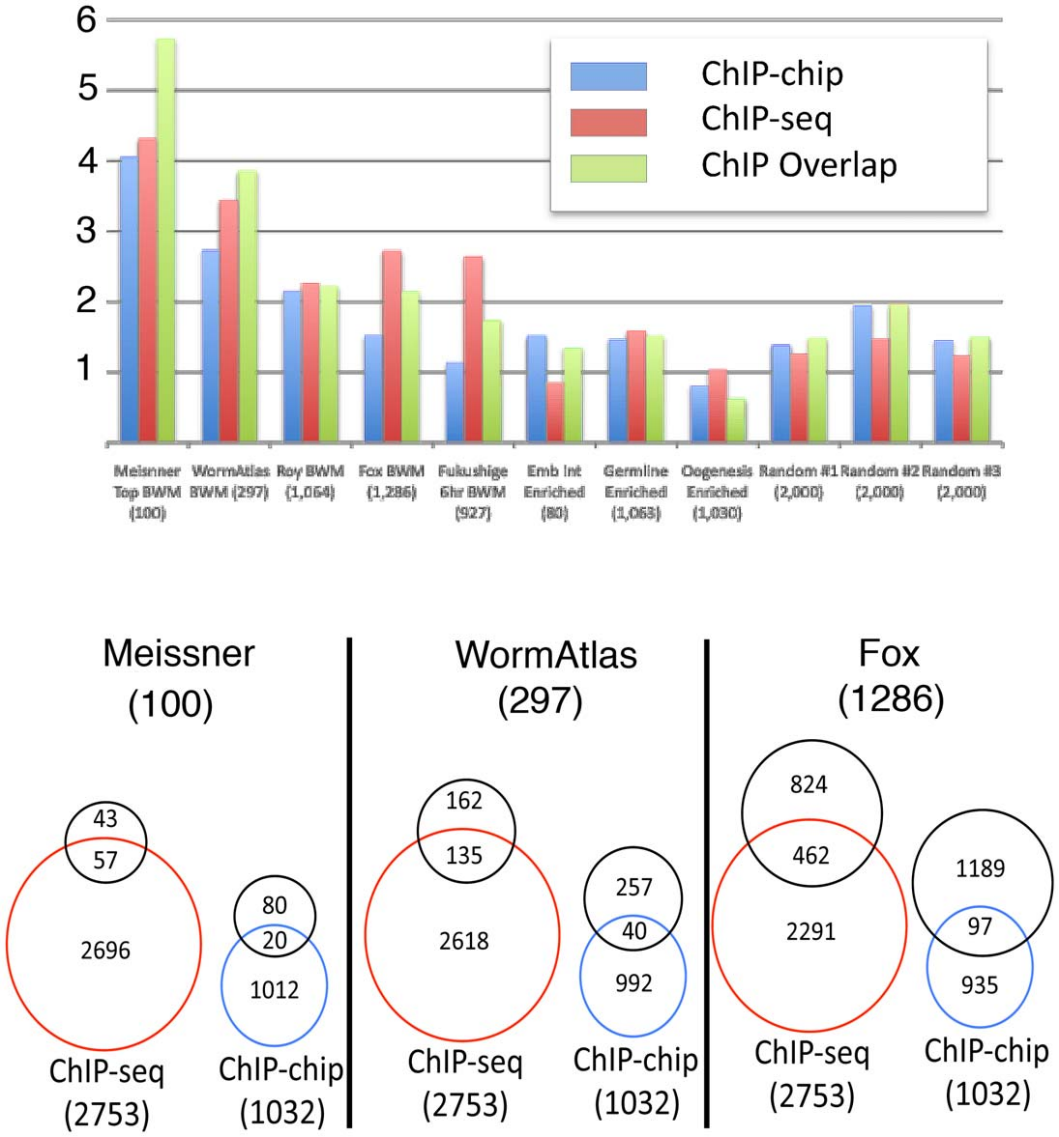
Comparison of ChIP-associated genes and bodywall muscle transcription. (Top) Bodywall muscle gene enrichment. Lists of peak-associated genes identified by HLH-1 ChIP-chip, ChIP-seq, or the overlap of both ChIPs were compared with lists of genes thought to be expressed and/or enriched in bodywall muscle cells during embryonic and/or post-embryonic development from previous studies (see text). As controls, lists for genes expressed in intestinal (Int), germline and oogenesis, as well as three random gene lists (see text), were also compared to the ChIP data. The fold enrichment for these comparisons relative to theoretical randomization (value of 1.0) is plotted for each list with the number of genes in each list noted parenthetically. The ChIP data (regardless of method) was significantly enriched for genes expressed and/or enriched in bodywall muscle compared to the random list controls. (Bottom) Bodywall muscle and ChIP gene data overlap. Overlap details on the data for three of the bodywall muscle gene lists as indicated. For each list, the number of overlapping genes in comparison to either the ChIP-seq or ChIP-chip is indicated by Venn diagrams. The total number of genes in each list is given parenthetically.

We compared our HLH-1 ChIP-chip and ChIP-seq candidate target gene lists with each of these previously identified bodywall muscle gene lists, normalizing to the number of genes in each list. As controls for these comparisons, we used three randomly generated sets of 2,000 genes each using an alphabetical sort of all genes from WormBase (WS195) data http://www.wormbase.org as well as lists of genes previously described as enriched in the adult germline (1,063 genes)[10], oogenesis (1,030 genes) [25], or embryonic intestine (80 genes) [12]. As shown in Figure 6, previously identified bodywall muscle enriched/expressed gene lists were over-represented in our ChIP data, with similar results for both HLH-1 ChIP-chip and ChIP-seq experiments. As expected, the most highly expressed genes in bodywall muscle, as assayed by muscle cell sorting and SAGE tag counts (top 100), were the most enriched in the ChIP data (Figure 6). Note that for these genes, the overlap of ChIP-chip and ChIP-seq data was also the highest, demonstrating that validation by the two independent ChIP methods provides higher confidence of bodywall muscle expression. For two of the bodywall muscle gene lists, the ChIP-seq data was significantly enriched whereas the ChIP-chip data was close to background. Each of these two muscle gene expression lists was derived from manipulated embryos assayed by expression microarrays. It is not obvious why such assays would result in the strong bias towards under-representation in the ChIP-chip data, although those experiments were looking at early onset, rather than persistent, expression in bodywall muscle. Among the control gene lists, one of the three random lists was slightly enriched in the ChIP data whereas the other two, along with the germline-related lists and the embryonic intestine list, were at background levels. We concluded that both experimental approaches to identify HLH-1 binding sites by ChIP resulted in associated candidate target genes that were enriched for either suspected or known bodywall muscle genes, suggesting HLH-1 activation of these targets is direct. We cannot exclude the possibility that HLH-1 also functions as a negative regulator of transcription in other somatic tissues.

Previous studies have suggested that the distribution of bodywall muscle genes throughout the genome is non-random, with evidence for clustering and under-representation of muscle genes on certain linkage groups, including the X chromosome [22]. We analyzed the chromosomal distribution of potential HLH-1 target genes identified by ChIP and found that the X chromosome had an over-, not under-, representation of such genes, with 13–18% of genes on autosomes versus 21–29% on the X chromosome. Restricting the analysis to only the 569 genes identified both by ChIP-chip and ChIP-seq, the X chromosome contained 34% of the genes, chromosomes I and V having a low of only 9–10%, and the remaining autosomes ranging between 14–17%. Inspection of the X-linked genes revealed no obvious pattern in distribution or over-represented process by GO analysis.

### HLH-1 ChIP interval validation in vivo is problematic

In order to assay the potential biological relevance of the HLH-1 binding sites identified by ChIP, we generated GFP reporter genes by placing HLH-1 bound interval sequences upstream of a *myo-2::gfp* minimal promoter. We, and others, have used the *myo-2::gfp* minimal promoter (Fire vector L3136) in the past as a robust assay for bodywall-specific transcriptional enhancer activity for test sequences [7], [26]. We selected 20 positive peak intervals identified by HLH-1 ChIP-chip, 17 of which overlapped with ChIP-seq called peaks. As shown in Table 1, these intervals were heavily biased towards association with known bodywall muscle expressed genes (18/20), varied in length between 350 and 2,333 bp and, with one exception, contained multiple E-box sequences. Surprisingly, only 6 of these 20 intervals (associated with the genes T28B11.1, c*ye-1, etr-1, let-60, unc-60*, and *ric-3*) were positive for bodywall muscle enhancer activity when introduced as stable extrachromosomal transgenes in an otherwise wild type background (Table 1; Figure S2). These six positive fragments had a wide range of E-boxes (4–13), were all detected by both ChIP-chip and ChIP-seq approaches, and had elevated peak scores with a value of 4.6 or greater in the HLH-1 ChIP-chip data. Interestingly, 12 of the 14 intervals that failed to direct bodywall muscle expression of the reporter gene were associated with genes known to be expressed in bodywall muscle. That is, the associated gene was expressed in bodywall muscle, however, the ChIP identified HLH-1 binding site interval was not sufficient by itself to drive expression of an enhancer test reporter gene in those cells. We concluded that no single metric associated with an HLH-1 positive interval reliably predicted in vivo bodywall muscle enhancer activity, although active intervals usually had higher peak scores, contained multiple E-box elements, and were detected by both ChIP-chip and ChIP-seq approaches.

**Table 1:** Reporter Gene Construct Details and Results.

		ChIP-chip Metrics							Evidence for BWM expression				
Cosmid	Gene	Pk Score	Pk Rank	Peak (bp)	E-box	Seq Pk	Enh^1^	hsHLH-1	Reporter	Antibody	Array	Atlas^2^	Ref
ZK867.1	*syd-9*	37.16	2	1864	9	YES	No	*	YES	YES	YES	*	[33]
T28B11.1	T28B11.1	35.66	3	1616	11	YES	YES	*	*	*	YES	*	[5]
T13H2.4	*pqn-65*	34.72	6	1136	6	YES	No	*	*	*	YES	*	[5]
C07A9.3	*tlk-1*	34.57	7	2272	4	YES	No	*	*	*	*	*	N/A
T05A10.1	*sma-9*	33.59	13	2333	17	YES	No	*	*	*	*	*	N/A
F33A8.3.1	*cey-1*	6.70	97	2022	6	YES	YES	*	*	*	YES	*	[5]
C01G6.1b.1	*aqp-2*	6.40	122	1890	13	YES	No	No	YES	*	YES	*	[34]
T01D1.2a.1	*etr-1*	5.35	236	2150	13	YES	YES	No	YES	*	YES	*	[35]
ZK792.6	*let-60*	5.09	284	910	5	YES	YES	*	YES	*	YES	*	[36]
C38C3.5b.1	*unc-60*	5.02	300	1343	4	YES	YES	*	*	YES	YES	*	[37]
C47E8.7.1	*unc-112*	4.74	361	1739	8	YES	No	No	YES	YES	YES	*	[38], [39]
T14A8.1	*ric-3*	4.69	372	1651	8	YES	YES	No	YES	*	YES	*	[40]
B0350.2a.2	*unc-44*	4.19	515	416	1	No	No	*	YES	*	YES	YES	[41]
F45E4.2.1	*plp-1*	4.19	531	905	4	YES	No	*	YES	*	YES	YES	[42]
K04H4.1b	*emb-9*	4.14	548	1551	9	YES	No	No	YES	YES	YES	YES	[41], [42]
F21H11.3.2	*tbx-2*	4.00	593	950	5	No	No	*	YES	*	YES	*	[43]
F55C7.7a	*unc-73*	3.95	612	680	5	YES	No	No	YES	YES	YES	YES	[41], [44]
F10E9.6b	*mig-10*	3.70	699	611	8	YES	No	*	YES	*	YES	YES	[41]
M88.5b	M88.5	3.39	848	867	4	No	No	*	YES	*	YES	YES	[41]
F31E3.1	*ceh-20*	2.88	1069	350	2	YES	No	No	YES	*	YES	*	[45]

1 Enh – Positive for bodywall muscle enhancer activity.

2 Atlas – Reported with bodywall muscle expression in WormAtlas.

It was possible that the failure of HLH-1 bound intervals to enhance bodywall muscle reporter gene expression in wild type animals was due to the relatively low levels of endogenous HLH-1 compared to the levels of heat shock induced HLH-1 or epitope-tagged HLH-1 used to identify potential binding sites by ChIP. To determine if that was the case, we introduced seven of the enhancer assay reporter constructs into a strain harboring the heat shock promoter driven HLH-1 transgene and assayed bodywall muscle enhancer activity with and without heat shock induction of HLH-1. The seven reporters tested included five that failed in the original enhancer assay in a wild type background (*emb-9, unc-112, ceh-20, aqp-2, unc-73*) and two that were positive for expression in wild type animals (*etr-1, ric-3*). All seven reporter constructs were injected, along with a rescuing *unc-119* plasmid, into the *hsp::hlh-1, unc-119* stain and non-Unc animals selected for the assay. None of the seven reporters showed a strong or reproducible response to heat shock induced HLH-1 when assayed at multiple times after induction in embryos, larvae, or adults (Table 1). To confirm that the heat shock promoter driving the cDNA encoding HLH-1 was still active in these strains, we assayed by Western blot the level of HLH-1 protein before and after heat shock in mixed stage embryos. All stains strongly induced HLH-1 to high levels within 3 hours after induction, as previously reported (Figure S3) [7]. We concluded that the ability of a genomic interval to bind HLH-1 by ChIP analysis was a poor metric for ascribing biological activity in vivo for the interval functioning out of its normal genomic context. This is particularly evident from the failure of heat shock induced HLH-1 to activate any of the enhancer reporter genes; this enhancer reporter resulted in strong, widespread, ectopic activity in embryos when used with a known HLH-1-responive enhancer in a previous study [7].

## Discussion

Our study was aimed at identifying the binding sites of HLH-1 genome-wide during embryogenesis while addressing two common problems associated with transcription factor ChIP assays; limited temporal-spatial patterns of expression and lack of suitable reagents for immunoprecipitation. These problems were addressed using two completely different methodologies. In one approach, we used antibodies directed against HLH-1 in combination with heat shock induced over expression of HLH-1 in transgenic animals with ChIP probes hybridized to a genome tiling array (ChIP-chip). As an alternative, we used an epitope-tagged (GFP) HLH-1 transgene that was ChIPed with anti-GFP antibodies and the immunoprecipitated DNA analyzed by next generation sequencing (ChIP-seq). Using these alternative approaches for the transcription factor HLH-1 in *C. elegans* embryos reveals similar genome-wide profiles for this master myogenic regulator. Although epitope-tagging has been used previously as a surrogate to interrogate binding site distributions in multiple systems, the use of heat shock induced over expression in vivo for such studies is novel. Despite concerns that over expression might result in a high rate of false positive binding sites, our data suggests that is not the case in *C. elegans*. Our results expand the repertoire of experimental approaches for ChIP studies to include options readily engineered in most biological systems.

This study provides valuable insights into HLH-1 biology in *C. elegans*. Both ChIP datasets demonstrate that HLH-1 can be detected just upstream of the TSS of thousands of potential target genes, that an E-box (CANNTG) is the dominant over-represented motif in these bound sequence intervals, and the genes associated with binding sites are enriched in those known to be expressed in bodywall muscle. The widespread localization of HLH-1 predominantly to promoter regions is consistent with the expectation that HLH-1 acts directly to regulate target gene expression. HLH-1 activity is likely mediated through direct binding to E-box sequences (CANNTG) in the promoter of target genes. The symmetry of the top returned E-box motifs is suggestive of homodimer binding, consistent with the fact that *C. elegans* bodywall myogenesis occurs in the absence of the E protein binding partner for the MRFs typically found in other biological systems [17]. The strong association of HLH-1 binding sites upstream of genes known to be expressed in bodywall muscle cells, including many structural genes, suggests that HLH-1 acts as a transcriptional activator of these genes. Thus, our data support a model in which HLH-1 homodimers direct bodywall myogenesis by directly binding to, and activating, many genes required for the development and function of muscle.

There were also several surprising aspects of our results from this study. We did not anticipate the large number of HLH-1 bound intervals and associated genes, up to half of all protein coding genes in *C. elegans* in the ChIP-seq data using default threshold values. Equally important was the lack of evidence for widespread, ectopic binding of HLH-1 after over expression in the ChIP-chip data. We found that only a small fraction (∼3.5%) of all possible E-boxes genome wide were identified by either our ChIP-chip or ChIP-seq (10^−6^) approaches, arguing for a high degree of selectivity for HLH-1 binding. A comparison of the HLH-1 ChIP data to that recently published for another transcription factor, PHA-4, also suggests highly selective binding (1). Although the peak overlaps between HLH-1 and PHA-4 embryonic datasets was slightly above random expectations, the associated gene overlap was at, or far below, random expectations for all pair wise comparisons. HLH-1 and PHA-4 target genes would not be expected to overlap in embryos as these two transcription factors are expressed in mutually exclusive tissues at this stage of development. Further studies are required to determine if the binding site specificity we observed is an intrinsic property of HLH-1 or reflects additional constraints, including required cooperativity with other factors or the limited accessibility to potential binding sites in the context of general or tissue-specific chromatin organization.

We were also surprised to find that neither of the ChIP methods identified a majority of the genes defined previously by various groups cataloging the bodywall muscle transcriptome. The best observed overlap (45%) was between the ChIP-seq (10^−6^) and the 297 WormAtlas bodywall muscle expression gene list. The inability to identify all bodywall muscle genes by mixed stage embryo ChIP technology is not completely unexpected because some bodywall muscle genes may be activated late in embryogenesis or post-embryonically. However, the discordance between the ChIP data and bodywall muscle transcriptome profiles also highlights the inherent flaws in experimental manipulation, current technologies employed for gene expression and ChIP studies, and bioinformatic treatment of the resulting data. At the level of gene expression, each of the several attempts to define the bodywall muscle transcriptome in *C. elegans* has generated a gene list, yet there is limited consensus among these. These differences arise due to many factors, including methodological efficiencies, life stage variability, and the use of various experimental platforms to assay expression. At the ChIP level, our results similarly highlight the variability associated with experimental and platform differences. For example, our ability to validate ChIP-seq peaks not detected by ChIP-chip, using the ChIP-chip reagents and methods demonstrates that our ChIP-chip data is under representing the HLH-1 binding sites. One likely explanation of this under representation is the limited dynamic range of chip hybridizations that makes the discrimination of signal and background more difficult compared to the more quantitative next generation sequencing approach. In addition, our ChIP-chip peak calls required consensus between three biological replicates, a stringency that may have eliminated true positives. The identification and validation of ChIP-chip HLH-1 binding sites that were not detected by ChIP-seq are more difficult to explain. Clearly, no single approach captures a clear and complete view of transcriptional systems suggesting a combination of approaches is likely needed to more fully understand the relationship of transcription factor DNA binding and gene expression.

Our inability to validate many HLH-1 bound sequences with in vivo assays for enhancer activity, even in the presence of heat shock induced over expression of HLH-1, was also unexpected. Although such reporter assays are fraught with caveats, they frequently can be successfully used to demonstrate enhancer function. It seems likely that many sites that bind HLH-1 alone in vivo may not be associated with transcriptional activity. Such sites may never be transcriptionally active, may require additional factors to cooperate for activation through associated cis-acting sequences, or may be sites of transcriptional repression. Regardless, the large number of binding sites makes the correlation of ChIP data with bodywall muscle transcriptomes an imperfect science, demonstrating clear overlaps between the datasets, but at levels that are insufficient to describe the network with any confidence.

A recent study of genome binding sites of the mammalian homolog of HLH-1, MyoD, found results very comparable to ours [27]. That study suggested that mouse MyoD binds approximately 60,000 sites on the autosomes, is associated with between 41–74% of all protein coding genes, is positively correlated with genes expressed during myogenesis, and that MyoD binding sites often fail in enhancer activity assays in tissue culture. These remarkably similar observations for MyoD in the mouse system to our results in *C. elegans* for HLH-1 suggest evolutionary conservation in the roles for this master regulatory transcription factor, both in activation of target muscle genes and in as yet to be determined functions genome-wide.

Our results suggest, as in mammals, that many of the binding sites for HLH-1 in *C. elegans* may be functionally inert or play a role not directly linked to transcriptional activation, such as modifying or maintaining chromosomal architecture necessary for a committed muscle cell fate. These alternate functions may underlie the non-random distribution of HLH-1 binding sites on the chromosomes, including a vast over-representation of the ChIP overlapping set on the X chromosome. The high number of genome-wide binding sites for HLH-1/MyoD presents an unanticipated challenge for the field in interpreting the increasing volumes of transcription factor ChIP data becoming available. An unexpectedly large number of binding sites have also been observed for several transcription factors in *Drosophila*, suggesting this is a general feature of these factors [28], [29], [30]. Clearly, the function of transcription factors, as well as the predictive power with respect to transcriptional activation or repression, will require the combination of profiles for multiple factors and chromatin modifications to be fully understood.

## Methods

### *C. elegans* strains

The following *C. elegans* strains were used: wild type (N2); the heat shock promoter driven *hlh-1* strains KM472 and KM267 [5]; a derivative of KM267 in which we introduced the selectable transformation mutation *unc-119 (ed3)* for testing enhancer reporter genes in the presence of over expressed HLH-1.

### Generating transgenic lines

A GFP-tagged *hlh-1* coding region in the context of a fosmid clone was introduced into animals by biolistic transformation using the *unc-119* selection marker, as previously described [31]. Stably segregating lines were selected over four generations prior to use in ChIP-seq experiments. Transgenic strains for assaying expression were generated using standard injection techniques with 10–100 ug of test plasmid and 50 ug of the selectable dominant *rol-6* plasmid pRF4. At least two stable transgenic lines were generated for each construct and expression in both adult and embryonic bodywall muscle lineages was determined. The 20 fragments corresponding to the selected peak sequences (Table 1) were amplified using *C. elegans* genomic DNA as template. The amplified fragments were inserted into the PstI site of the L3136 plasmid upstream of the basic promoter reporter construct *P_myo2_-gfp-lacZ* to make a series of reporter constructs. All reporter gene construct sequences were confirmed by DNA sequencing. PCR primers for all reporter constructs are listed in Table S4.

### Chromatin Immunoprecipitation (ChIP) Assays

The ChIP assay was conducted as described previously [7]. The *hsp::hlh-1* transgenic strain KM267 was used in this assay. For the ChIP-chip assay, ChIP DNA from heat shock *hsp::hlh-1* transgenic lines after cross-linking and immunoprecipitation with antibody against the HLH-1 was amplified with the GenomePlex whole genome amplification kit (Sigma-Aldrich) according to the protocols provided by Nimblegen. Immunoprecipitated DNA with chicken anti-HLH-1 (experimental) or pre-immune chicken serum (control) samples was prepared in biological triplicates. Nimblegen *C. elegans* tiling array contain 385,000 oligonucleotides covering the *C. elegans* genome at a resolution of 50 bp with 36 bp gaps between probes. ChIP-chip hybridization and scanning were done by Nimblegen. ChIP-seq assays were conducted using the modENCODE pipeline as previously described [1].

### Data analysis

Quantile normalization, peak detection, gene annotation, peak distribution was done using the CARPET tiling analysis software package [8]. HLH-1 bound peaks and interval sequences were identified using the following parameters in the notator function: analysis type, p-value; percentile value, 0.95; -log p-value cutoff, 13; minimal number of probes, 5; max distance between two probes, 100; min distance between two peaks, 200; window length, 500; promoter definition (bp), −2000; annotation priority, gene. We employed two different software packages to compare the overlap of peak calls between datasets, with very similar results from each. The CARPET package [8] Com&Easy function was used with the following settings:parameter set: Principal table, variable p-value cut off for the ChIP-seq peaks; Secondary table, ChIP-chip peaks; Analysis type, common; Coordinate common, Principal Table. Alternatively, we used Galaxy software [9] to compare peak calls. Galaxy was used to generate the data shown in Table S2. MDscan [14] was used for the Motif search.

### Confirmation of ChIP-chip binding regions using qPCR

Primers were designed for HLH-1 ChIP enriched genomic DNA regions using Primer3 (http://frodo.wi.mit.edu/primer3/) [32]. Real-time quantitative PCR (qPCR) was performed on the ABI 7900HT sequence detection system using the Power SYBR Green PCR master mix (Applied Biosystems). Primers and associated genes for the qPCR amplicons are listed in Table S4.

## Supporting Information

Figure S1 Genome browser view comparison of ChIP data. An Integrated Genome Browser (IGB) view of the ChIP data for a 1,000 kb region of LGII of *C. elegans* shows the peak intervals at top (blue boxes) relative to gene coding regions on the plus and minus coding strands below (green boxes). ChIP-seq intervals using both default and e^−6^ thresholds are shown for comparison to the ChIP-chip intervals. Note the similarity in patterns among ChIP datasets with the ChIP-chip intervals constituting an increasingly smaller subset as the ChIP-seq data threshold is reduced.
(TIF)

Figure S2 The HLH-1 bound interval upstream of *etr-1* drives bodywall muscle expression. The 2,150 bp interval identified by HLH-1 ChIP-chip and ChIP-seq methods was fused to a reporter gene used to assay bodywall muscle activity and introduced into a wild-type background by transgenesis. This genomic fragment was sufficient to drive bodywall muscle expression of the reporter in embryos, larvae, and adults.
(TIF)

Figure S3 Heat shock induction of HLH-1. Total embryonic lysates from wild type animals (Cntrl) or strains harboring a heat shock inducible *hlh-1* cDNA transgene (paired lanes 1–7) were assayed by Western blot probed with chicken anti-HLH-1 antibody. Lysates were prepared from embryos of gravid adults three hours after mock (−) or heat shock treatment (+) and equal amounts of total protein was loaded in each lane. All transgenic strains show strong induction of HLH-1 in response to heat shock. M is molecular size marker. Samples 1 & 2 are the heat shock *hlh-1* strains, KM267 & KM472, respectively, which have been previously characterized (Lei et al., 2009). Samples 3–7 are embryo extracts from strains harboring both the heat shock *hlh-1* transgene and reporter genes of amplicons associated with the following genes: sample 3: *aqp-2*, sample 4: *etr-1*, sample 5: *ric-3*, sample 6: *ceh-20*, sample 7: *emb-9*. Arrows indicate full-length HLH-1.
(TIF)

Table S1 ChiP-chip peak call data.
(XLS)

Table S2 Gene list calls for ChiP-chip, ChIP-seq, and the overlap of each method.
(XLS)

Table S3 ChiP-seq (e^−6^) peak call data.
(XLS)

Table S4 Primer sequence information.
(XLS)
