# Supplementary figures and images for: A Widespread Distribution of Genomic CeMyoD Binding Sites Revealed and Cross Validated by ChIP-Chip and ChIP-Seq Techniques
Source: PLoS One. 2010 Dec 29;5(12):e15898. doi: 10.1371/journal.pone.0015898 (PMC3012110; doi:10.1371/journal.pone.0015898)

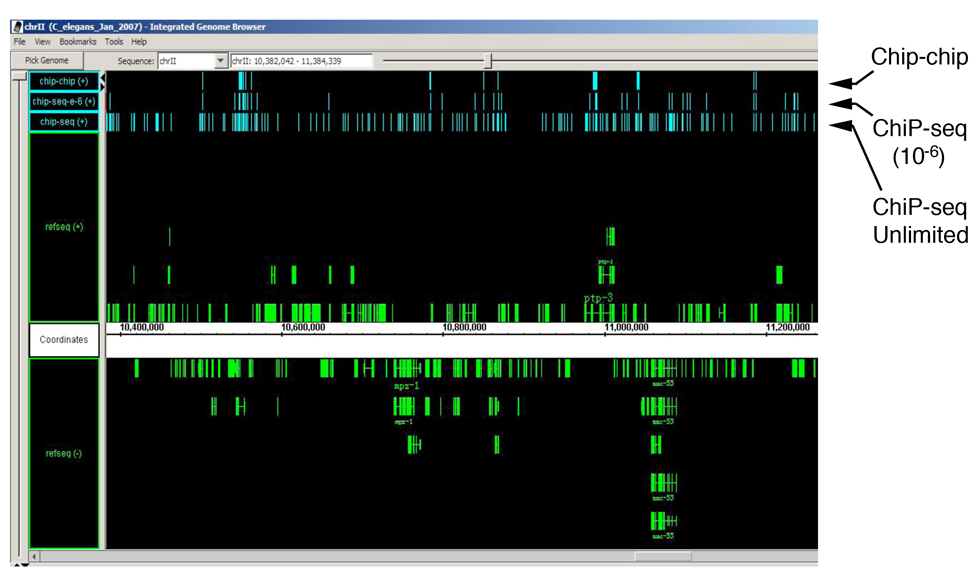

Supplement: Figure S1 — Genome browser view comparison of ChIP data. An Integrated Genome Browser (IGB) view of the ChIP data for a 1,000 kb region of LGII of C. elegans shows the peak intervals at top (blue boxes) relative to gene coding regions on the plus and minus coding strands below (green boxes). ChIP-seq intervals using both default and e−6 thresholds are shown for comparison to the ChIP-chip intervals. Note the similarity in patterns among ChIP datasets with the ChIP-chip intervals constituting an increasingly smaller subset as the ChIP-seq data threshold is reduced. (TIF) [file pone.0015898.s001.tif]

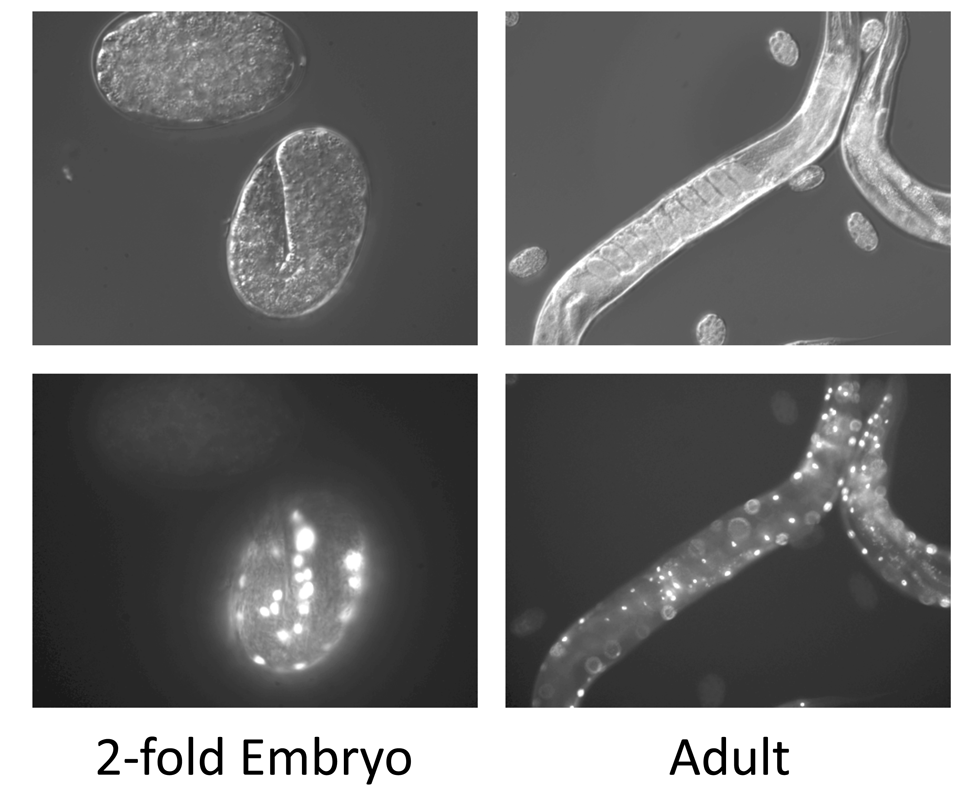

Supplement: Figure S2 — The HLH-1 bound interval upstream of etr-1 drives bodywall muscle expression. The 2,150 bp interval identified by HLH-1 ChIP-chip and ChIP-seq methods was fused to a reporter gene used to assay bodywall muscle activity and introduced into a wild-type background by transgenesis. This genomic fragment was sufficient to drive bodywall muscle expression of the reporter in embryos, larvae, and adults. (TIF) [file pone.0015898.s002.tif]

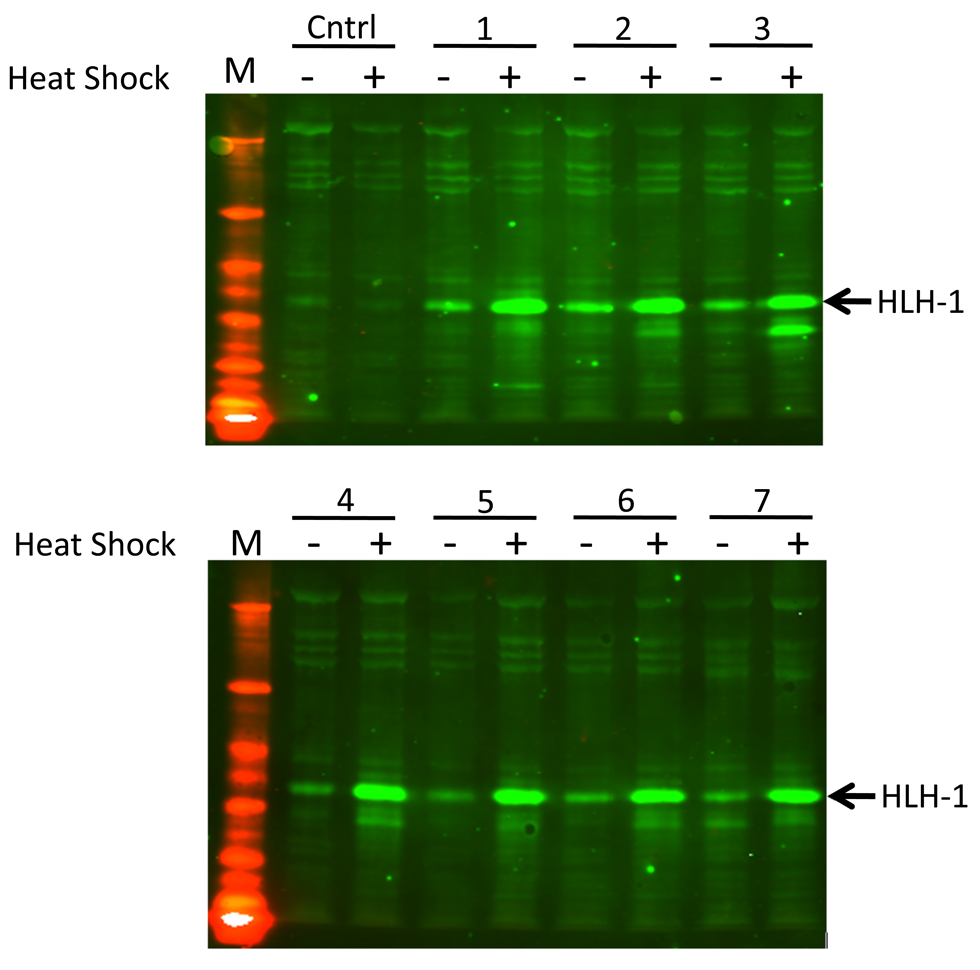

Supplement: Figure S3 — Heat shock induction of HLH-1. Total embryonic lysates from wild type animals (Cntrl) or strains harboring a heat shock inducible hlh-1 cDNA transgene (paired lanes 1–7) were assayed by Western blot probed with chicken anti-HLH-1 antibody. Lysates were prepared from embryos of gravid adults three hours after mock (−) or heat shock treatment (+) and equal amounts of total protein was loaded in each lane. All transgenic strains show strong induction of HLH-1 in response to heat shock. M is molecular size marker. Samples 1 & 2 are the heat shock hlh-1 strains, KM267 & KM472, respectively, which have been previously characterized (Lei et al., 2009). Samples 3–7 are embryo extracts from strains harboring both the heat shock hlh-1 transgene and reporter genes of amplicons associated with the following genes: sample 3: aqp-2, sample 4: etr-1, sample 5: ric-3, sample 6: ceh-20, sample 7: emb-9. Arrows indicate full-length HLH-1. (TIF) [file pone.0015898.s003.tif]
